# Apparent Diffusion Coefficient Predicts Pathology Complete Response of Rectal Cancer Treated with Neoadjuvant Chemoradiotherapy

**DOI:** 10.1371/journal.pone.0153944

**Published:** 2016-04-21

**Authors:** Yuan-Gui Chen, Ming-Qiu Chen, Yu-Yan Guo, Si-Cong Li, Jun-Xin Wu, Ben-Hua Xu

**Affiliations:** 1 Department of Radiation Oncology, Fujian Medical University Union Hospital, Fuzhou City, Fujian Province, P.R. China; 2 Department of Radiation Oncology, University of Nebraska Medical Center, Omaha, NE, United States of America; 3 Department of Radiation Oncology, Fujian Provincial Cancer Hospital, Fuzhou City, Fujian Province, P.R. China; Instituti Ospitalieri di Cremona, ITALY

## Abstract

**Objective:**

To evaluate the predictive value of the apparent diffusion coefficient (ADC) for pathologic complete response (pCR) to neoadjuvant chemoradiotherapy (NCRT) in locally advanced rectal cancer.

**Methods:**

A total of 265 patients with rectal adenocarcinoma, whole Diffusion-Weighted MRI (DWI-MRI) images, clinically stage II to III (cT3-4 and/or cN+) and treated with NCRT followed by TME were screened. Fifty patients with pCR and another 50 patients without pCR with similar clinical charcacters and treatment regimens were selected for statistical analysis. All the patients’ pre-CRT and post-CRT average ADC values were calculated from the coefficient maps created by DWI-MRI and recorded independently. The difference in the ADC values between the pCR and non-pCR was analyzed by the Mann-Whitney U test. The cut-off ADC value of the receiver operating characteristic (ROC) curve with pCR was then established.

**Results:**

The mean pre- and post-ADC values in all patients, and in pCR patients and non-pCR patients were 0.879±0.06 and 1.383±0.11, 0.859±0.04 and 1.440±0.10, 0.899±0.07 and 1.325±0.09 (×10^-3^mm^2^/s), respectively. The difference between the pre- and post-ADC values in all patients, pCR patients, and non-pCR patients were considered to be statistically significant. The pre-ADC value was significantly lower in the pCR patients than in the non-pCR patients (*p* = 0.003), whereas the post-ADC values were significantly higher in the pCR patients than in the non-pCR patients. The percentage increase of the ADC value (ΔADC%) in the pCR and non-pCR patients were 68% and 48% respectively (*p*<0.001). The ROC curves of the cut-off value of the pre-CRT patient ADC value was 0.866×10^-3^mm^2^/s. The AUC, sensitivity, specificity, PPV, NPV, and accuracy of diagnosing pCR were 0.670 (95% CI 0.563–0.777), 0.600, 0.640, 60%, 60%, and 60%, respectively. The cut-off value of ΔADC% was 58%. The corresponding AUC, sensitivity, specificity, PPV, NPV, and accuracy of diagnosing pCR were 0.856 (95% CI 0.783–0.930), 0.800, 0.760, 76.9%, 79.2%, and 78%, respectively.

**Conclusions:**

DWI-MRI technology can be efficient for predicting pCR for LARC after NCRT. Although the mean pre-CRT ADC value and the ΔADC% are moderate predictors for pCR, the latter would be more accurate.

## Introduction

Rectal cancer is the tenth most common cancer in China[1], and most of patients are at a locally advanced stage at initial diagnosis. Neoadjuvant chemoradiotherapy (NCRT) combined with total mesorectal excision (TME) has become the standard treatment for locally advanced rectal cancer (LARC)[2,3].

Compared with postoperative chemoradiotherapy (CRT), NCRT is considered to have less acute toxicity, better local control, and higher rates of sphincter preservation[4]. In addition, patients achieving a pathologic complete response (pCR) after NCRT will benefit from better long-term survival than non-pCR patients and this may change the treatment strategy[5,6]. Actually, only some of the patients (11–27% reported) experienced a pCR after NCRT[7]. How to predict pCR in rectal cancer patients treated with NCRT by easy clinical merthods has not been determined.

Magnetic Resonance Imaging (MRI) has better soft tissue discrimination than Computed Tomography Imaging (CT), it provides more information about the pelvus than Endorectal Ultrasound (EUS) in patients treated with radiotherapy [8] and was recommended as one essential examination for clinical staging in rectal cancer[9]. With rapid technological developments of MRI, the Diffusion-Weighted MRI (DW-MRI), which depends on the microscopic mobility of water, can provide macromolecular and microstructural information prior to anatomical changes[10]. These are given in terms of the apparent diffusion coefficient (ADC), and DW-MRI is recommended as a clinical method for cancer imaging of biomarkers in many types of tumors[11]. Several studies have shown that it is feasible to use the ADC value to predict the response to NCRT in rectal cancer patients[12–14]. However, the studies of the ADC value predicting pCR as an endpoint after NCRT are few and the results are still controversial and require further investigation [15].

In the present study, the clinical data of LARC patients treated with NCRT followed with TME in our hospital were collected and retrospectively reviewed. The ADC values of patients with pCR after NCRT were evaluated and analyzed with non-pCR patients to provide a reference for accurately predicting prognosis.

## Materials and Methods

### Patient selection

This retrospective analysis was approved by the Fujian Medical University Union Hospital Institutional Review Board (No. 2013KY012). All patients completed informed consent prior to treatment and all information had been anonymized and de-identified prior to its analysis.

From January 2011 to July 2013, a total of 265 patients with pathologically confirmed rectal adenocarcinoma, whole DWI-MRI imagines, clinical stage II to III disease (cT3-4 and/or regional lymph-node positive), and treated with NCRT followed by TME were screened. Of these, 50 patients achieved pCR after NCRT. Another 50 patients without pCR (non-pCR patients) were selected from the remaining 215 patients for comparative statistical analysis in the current study. The non-pCR patients were similar to the pCR patients in gender, age, pretreatment T stage, pretreatment Clinical N, pretreatment tumor maximal thickness, pretreatment tumor long-axis diameter, interval between CRT and surgery, radiation dose of gross tumor volume, and chemotherapy, including the concurrent and intensive chemotherapy, regimens and cycles. The pretreatment CEA levels was one of the strong predictors for a response to NCRT reported in our previous study [16], and the pretreatment CEA level was the only significant difference between the pCR and non-pCR groups in the current study. The clinical characteristics are summarized in Table 1.

**Table 1:** The patients’ clinical characteristics.

Clinical characteristics	pCR	non-pCR
Average age, yrs	55.5 (31–68)	54.5 (35–69)
Gender		
male	34 (68%)	34 (68%)
female	16 (32%)	16 (32%)
Clinical T stage pre-CRT		
T2	5 (10%)	5 (10%)
T3	16 (32%)	16 (32%)
T4	29 (58%)	29 (58%)
Clinical N pre-CRT		
Positive	47 (94%)	47 (94%)
Negative	3 (6%)	3 (6%)
Concurrent Chemotherapy regimen		
CAPOX	42 (84%)	42 (84%)
Other regimen	18 (16%)	18 (16%)
Intense chemotherapy		
Yes/No	34/16	33/17
Average CEA level pre-CRT (ng/mL)*	6.08±6.68	21.85±62.34
Average CA19-9 pre-CRT (U/mL)	32.12±98.61	35.9±68.95
Average tumor maximal thickness pre-CRT (cm)	1.75±0.54	1.77±0.48
Average long-axis diameter pre-CRT (cm)	5.38±1.78	5.46±2.08
Average distance to the anus (cm)	4.91±1.68	5.20±1.49
Average interval between CRT and surgery (week)	8.58±1.27	8.31±1.39
Radiation dose of gross tumor volume (cGy)	5027.20±18.85	5029.60±17.72

CEA = Carcino Embryonic Antigen; CA19-9 = carbohydrate antigen,

* *p* = 0.021.

### Treatment

All patients were simulated on a computed tomography (CT) simulator. The definition of the clinical target volume (CTV) and the gross tumor volume (GTV) was published previously.[17] The planning target volume (PTV) was defined as an additional 1.0cm beyond the scope of the CTV and the GTV.

Fifty-four patients received three fields (two laterals with 15MV and one posterior with 6MV) conformal radiotherapy with a dose prescription of 45Gy in 25 fractions to the CTV and 5.4Gy in three fractions boosted to the GTV. The other 46 patients received five fields (6MV) IMRT with dose of 45Gy to the CTV and 50Gy to the GTV in 25 fractions [17].

All patients received concurrent chemotherapy with a 5-FU-based regimen. Of which, 84 patients were administrated with the CAPOX regimen. After the completion of NCRT, one or two courses of intensive neoadjuvant chemotherapy with a regimen of Folfox4 or CAPOX were conducted in 67 patients during the interval to TME.

The TME was performed at about 6–12 weeks after the completion of NCRT and at least two weeks after the completion of intense neoadjuvant chemotherapy.

### Diffusion-Weighted MRI

All patients received MRI scanning with a 3.0T superconducting MRI (Magnetom Trio Tim, SIEMENS Medical Systems, Germany), one week before the start of NCRT and within one week before surgery.

A 32-channel phased array body coil was used as the receiver coil. All scans were performed with patients in the supine position on a flat tabletop with no bowel preparation or taking antispasmodic drugs. The MRI protocol consisted of transverse T1-weighted, T2-weighted images, 3D-VIBE as well as transverse diffusion-weighted MRI. Axial DWI images were obtained using the single-shot echo-planar imaging technique [TR3900ms, TE40.3ms (b = 50s/mm^2^), TE53.8ms (b = 400s/mm^2^), TE76.7ms (b = 800s/mm^2^)]. Images were reconstructed with a 144×192 matrix, a slice thickness of 5 mm, and a slice gap of 1 mm. The total acquisition time for DWI was approximately 4–5 minutes.

**Fig 1:**
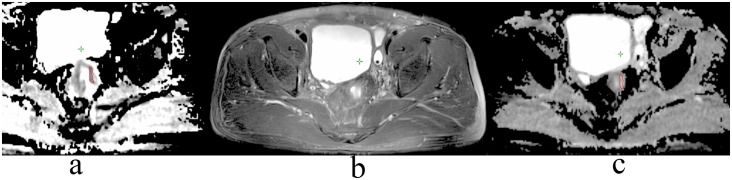
Delineation of ADC without positive tumor after NCRT. a. Delineation of the tumor on an ADC image before NCRT. b. No tumor could be found on T2WI after NCRT. c. Take the normal intestine in the same location as a measurement area because of the vanished tumor in the ADC image after NCRT.

The ADC maps were generated from the diffusion-weighted MR images with b-values of 800s/mm^2^. The ADC images were analyzed and measured on a Leonado workstation. With reference to the location and morphological characteristics of the lesion on the high-resolution T2WI, DWI, and 3D-VIBE scanning, the tumor was demonstrated at a relatively low signal intensity (SI) compared with normal intestine on ADC images (b = 800s/mm^2^). By excluding the necrotic or cystic portions inside the tumor, the tumor area at the layer where it had the maximal thickness was delineated as a region of interest (ROI). In order to make it closer to the ADC value of the whole tumor, the ADC value on both the above and below layers was also measured. After CRT, the ROI of the same anatomical location on the ADC images was contoured. If the tumor completely vanished after NCRT and no abnormal signal could be found on the ADC image, the normal intestine in the same location on the second image, with reference to the ROI pre-CRT, would become a measurement area (Fig 1)

The percentage increase of the ADC value (ΔADC%) defined as;

(Average post-CRT ADC value - Average pre-CRT ADC value) / Average pre-CRT ADC value × 100%,

were calculated. The value of the percentage increase was an indicator used to assess the impact of CRT on the change of ADC values. The statistical indication accuracy of the percentage increase values was quantitatively evaluated by use of the positive predictive value (PPV), negative predictive value (NPV), and predictive accuracy.

### Pathology

All of the tumor resection specimens were assessed by two experienced pathologists in accordance with the 7th edition of the AJCC Cancer Staging Manual[18]. A pCR was defined as complete tumor regression with only fibrotic tissues remaining. Otherwise, the cases were identified as non-pCR.

### Statistical Analysis

The data were analyzed with SPSS software, version 18.0 (SPSS, Inc., Chicago, IL, USA). The differences of the ADC value between before and after NCRT (pre-ADC and post-ADC) and ΔADC% were tested by the Mann-Whitney U test. Diagnostic performance for predicting pCR was evaluated using receiver-operating characteristic (ROC) curve analysis. Under the assumption of the cut-off value suggested by ROC curve analysis, the sensitivity, specificity, accuracy, positive predictive value (PPV), and negative predictive value (NPV) were calculated with the McNemar’s test. A two-tailed P value of less than 0.05 was considered statistically significant.

## Results

The mean pre- and post-ADC values in all patients, in the pCR patients, and in non-pCR patients were 0.879±0.06 and 1.383±0.11, 0.859±0.04 and1.440±0.10, 0.899±0.07 and 1.325±0.09 (×10^-3^mm^2^/s), respectively. The difference between the pre- and post-ADC values in all patients, pCR patients, and non-pCR patients were statistically significant (Table 2).

**Table 2:** The difference between the pre- and post-ADC values.

patients	pre-ADC value (×10^-3^mm^2^/s)	post-ADC value (×10^-3^mm^2^/s)	p
All	0.879±0.06	1.383±0.11	<0.001
pCR	0.859±0.04	1.440±0.10	<0.001
Non- pCR	0.899±0.07	1.325±0.09	<0.001

**Fig 2:**
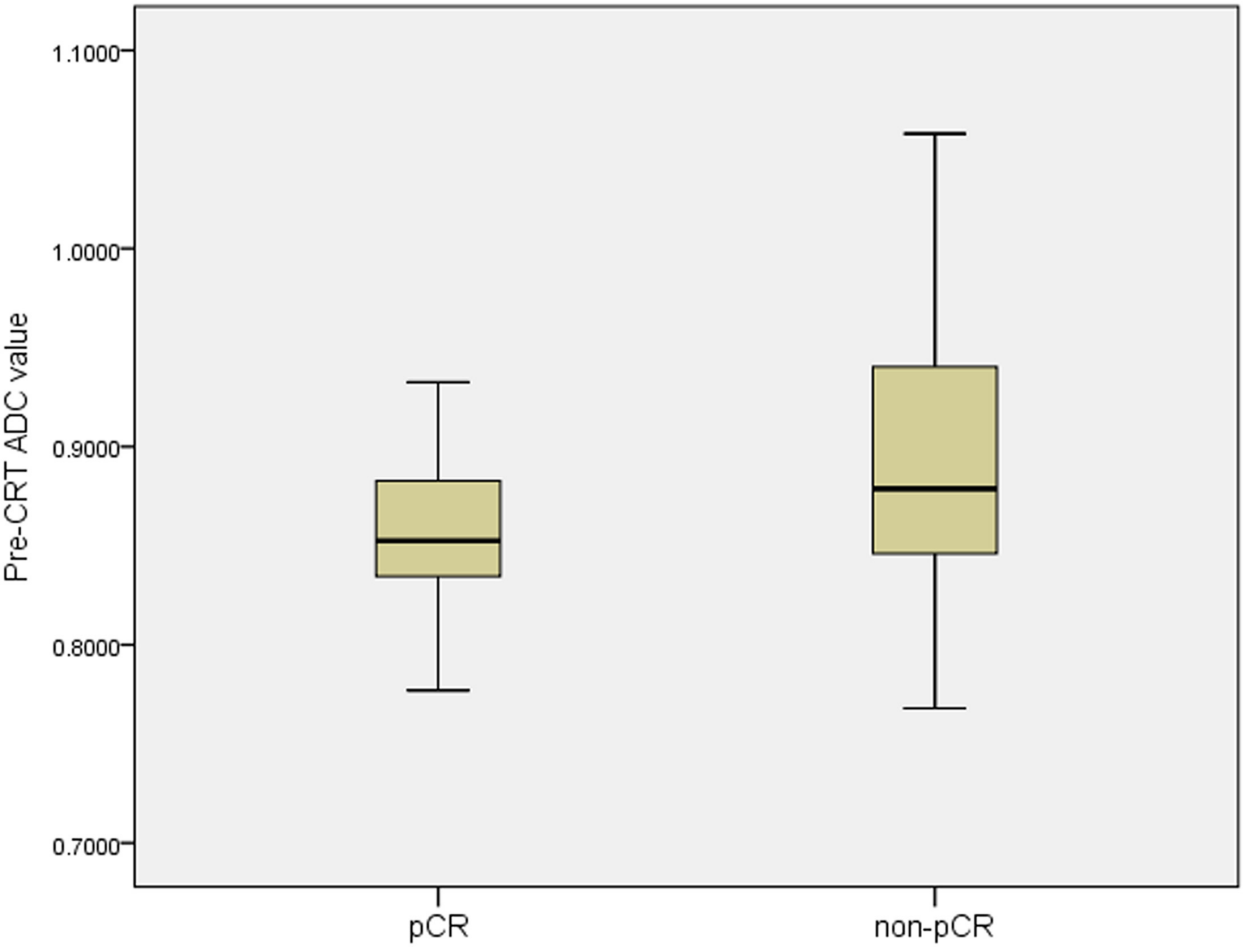
The mean value of pre-CRT ADC between the pCR and the non-pCR. The mean pre-CRT ADC value in the pCR group was significantly lower than that in non-pCR group (0.859±0.04×10^-3^mm^2^/s vs. 0.899±0.07×10^-3^mm^2^/s, p = 0.003).

**Fig 3:**
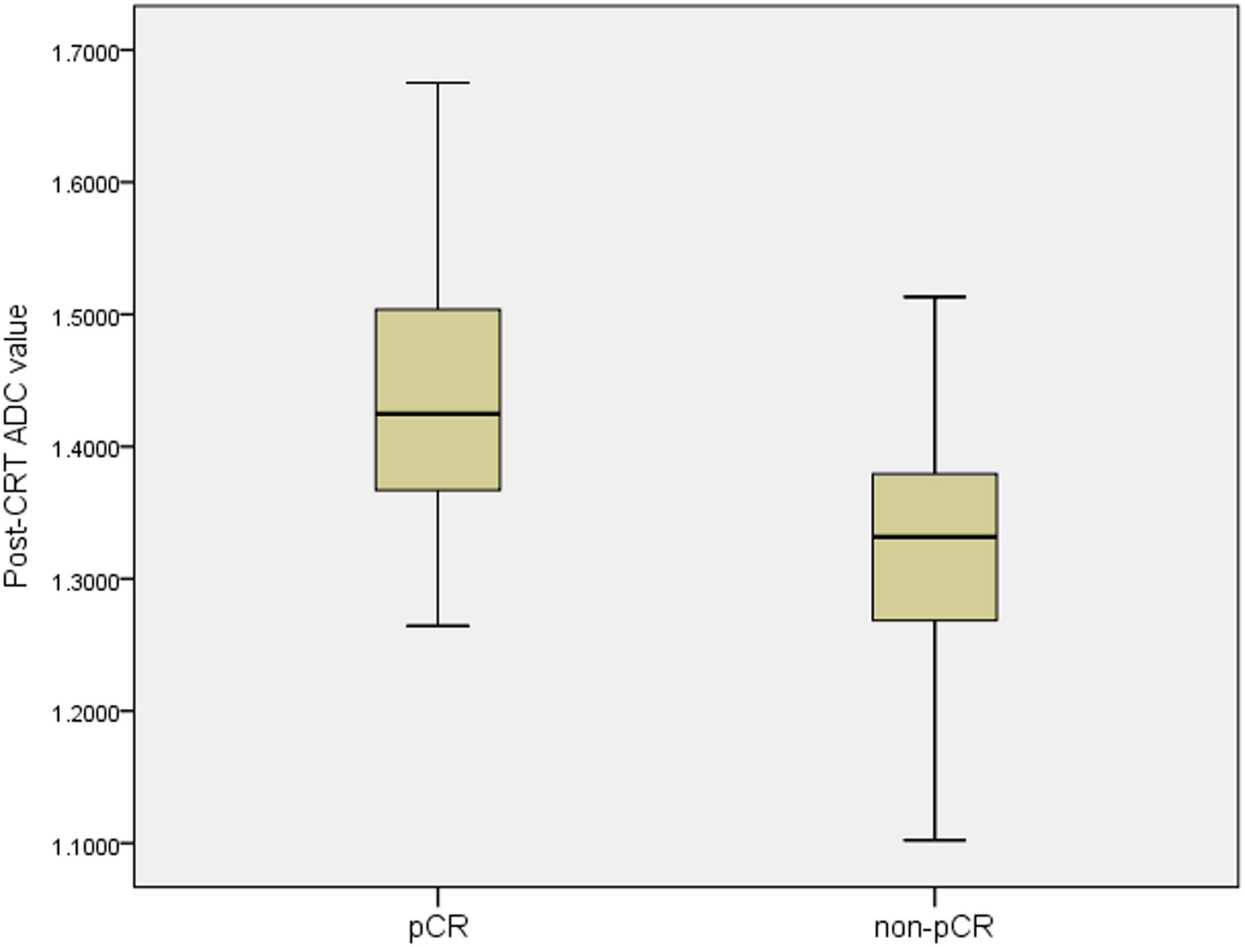
The mean value of post-CRT ADC between the pCR and the non-pCR. The mean post-CRT ADC value in the pCR group was significantly higher than that in non-pCR group (1.440±0.10×10^-3^mm^2^/s vs. 1.325±0.09×10^-3^mm^2^/s, p<0.001).

The pre-ADC value was significantly lower in the pCR patients than in the non- pCR group (*p* = 0.003, Fig 2), whereas the post-ADC value were significantly higher in the pCR than in the non-pCR patients (*p*<0.001, Fig 3). The ΔADC% in the pCR and non-pCR groups were 68% and 48% respectively, with significant heterogeneity (*p*<0.001).

**Fig 4:**
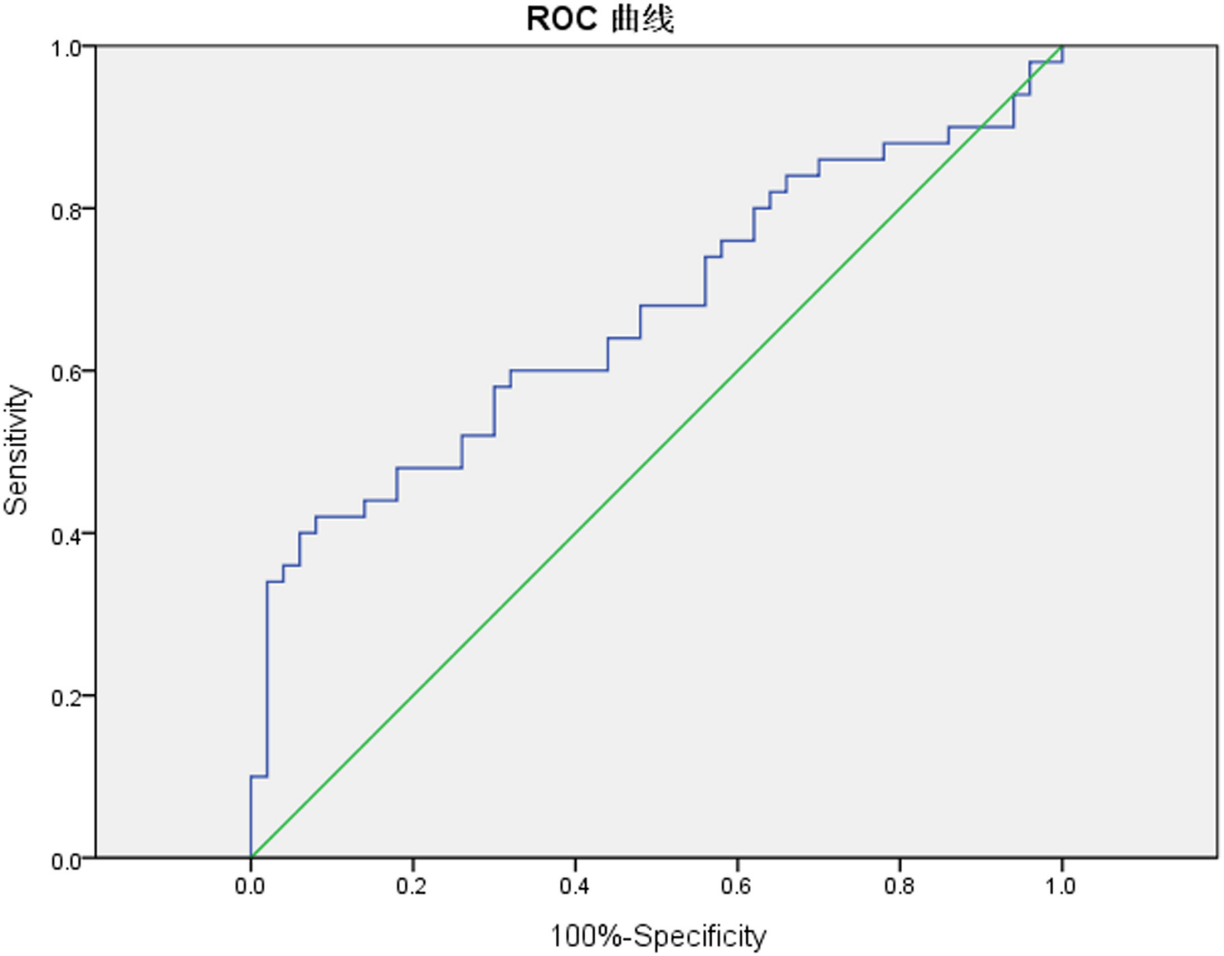
The ROC curves of mean pre-CRT ADC value to predict pCR. The ROC curves showed that the cut-off value of pre-CRT ADC value at the closest point to the top left corner was 0.866 ×10^-3^mm^2^/s. The sensitivity and specificity of diagnosing pCR were 0.600 and 0.640, respectively. And the AUC value equaled 0.670 (95% CI 0.563–0.777).

**Fig 5:**
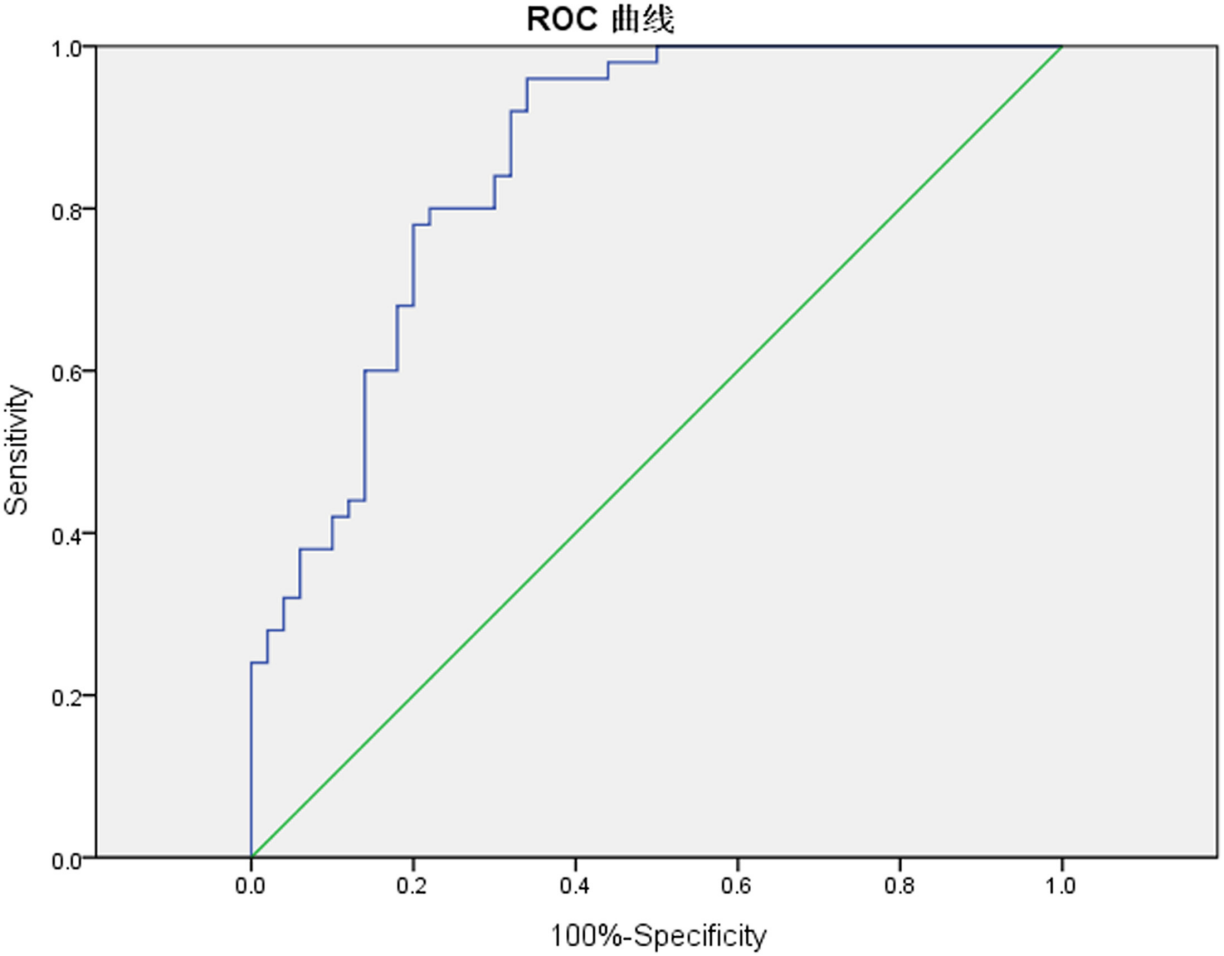
The percentage increase values of ADC to predict pCR. The cut-off value of the ADC percentage increase values at the closest point to the top left corner was 58%. The corresponding sensitivity and specificity of diagnosing pCR were 0.800 and 0.760, respectively. And the AUC value was 0.856 (95% CI 0.783–0.930).

The ROC curves were also plotted by using the pre-CRT ADC value and ΔADC. The ROC curves showed that the cut-off value of the pre-CRT ADC value was 0.866 ×10^-3^mm^2^/s (Fig 4). For diagnosing a pCR the AUC was 0.670 (95% CI 0.563–0.777), sensitivity was 0.600, specificity 0.640, PPV 60%, NPV 60%, and accuracy 60%. The cut-off value of ΔADC% was 58% (Fig 5). The corresponding AUC was 0.856 (95% CI 0.783–0.930), sensitivity 0.800, specificity 0.760, PPV 76.9%, NPV 79.2%, and accuracy of diagnosing pCR 78%. There was a higher accuracy when we take the ΔADC% as the predictive indicator (*p*<0.001), detailed in Table 3.

**Table 3:** Difference between Pre-CRT ADC and ΔADC% for prediction of pathological complete response.

Items	Pre-CRT ADC	ΔADC%	Χ^2^
Cut-off value	0.866×10^-3^mm^2^/s	58%	
AUG (95% CI)	0.670 (0.563–0.777)	0.856 (0.783–0.930)	
Sensitivity	0.60	0.80	
Specificity	0.64	0.76	
PPV	60% (30∕50)	76.9% (40∕52)	
NPV	60% (30∕50)	79.2% (38∕48)	
Accuracy	60% (60∕100)	78% (78∕100)	*p*<0.001

## Discussion

Dzik-Jurasz had first reported that the DW-MRI could be utilized as an imaging biomarker predicting the response to chemotherapy or chemoradiotherapy in rectal cancer patients[19]. In his study, the mean pre-ADC value before chemotherapy or chemoradiotherapy was less than the mean post-ADC value. The current study showed that an increase of the ADC value after NCRT in all patients, pCR patients, and non-pCR patients and the difference between pre- and post-treatment were statistically significant.

In previous studies, the lower pre-CRT ADC value used as a predictor for pCR is not conclusive. Kim [20] and Genovesi [21] had reported that the pre-ADC value in the pCR group did not differ significantly compared with the non-pCR group. However, Jung [22] showed that the pre-CRT ADC of the histopathological responders was significantly lower than that of the histopathological non-responses (*p* = 0.034). Sun et al. [23] showed that the low pre-ADC value in rectal carcinoma correlated with a good response to CRT (*p* = 0.013). In our study, the pre-CRT ADC value in pCR was significantly lower than non-pCR (0.859±0.04 vs. 0.899±0.07, *p =* 0.003). The pre-CRT ADC value may be a potential predictor for the pCR response to the NCRT.

There was a significant difference between the pCR group and the non-pCR group in the post-CRT ADC in our study. But the DWI currently used in the clinic limited the imaging resolution, which can cause a difficulty in accurately drawing a ROI for a regressed small tumor, especially in the pCR group. There might be a limitation for obtaining an outstanding diagnostic performance for predicting pCR by analysis of post-CRT ADC alone [15]. Although the results of the current study indicate that the post-CRT ADC value was significantly higher, it could not be used as a potential predictor for pCR, similar to what Intven et al. reported [24].

**Fig 6:**
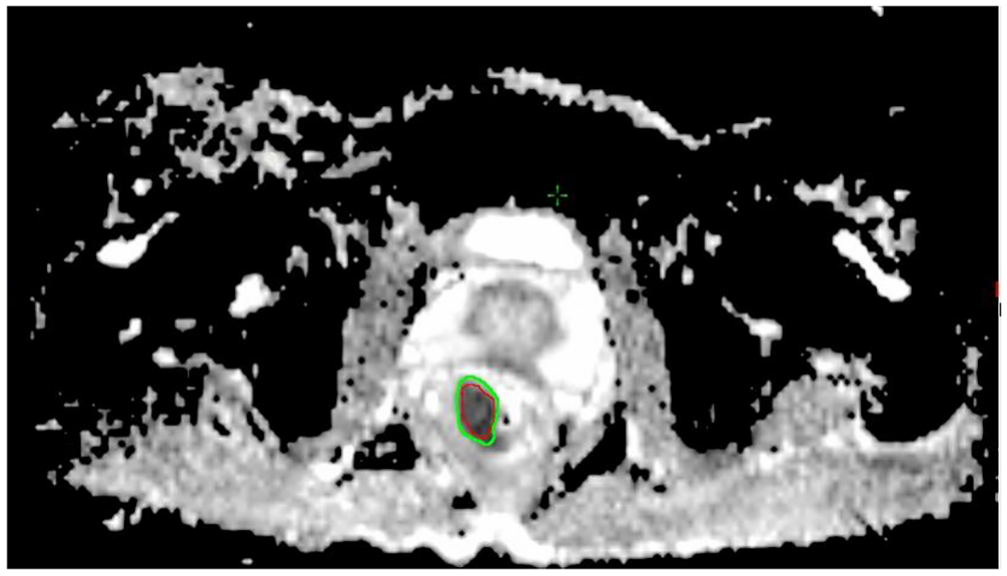
The deviation of the ROI on the mean ADC value. The mean ADC value of the red ROI and the green ROI were 0.801×10^-3^mm^2^/s and 0.911×10^-3^mm^2^/s, respectively. The difference of absolute value was only 0.11×10^-3^mm^2^/s, but the difference of relative value achieved 13.7%.

The measurement of the ADC value may be affected by the imaging quality, the spatial resolution of DWI images, and even a slight difference of the size and position of the ROI (Fig 6), and the relative value may be better for evaluation than the absolute value. Genovesi et al [21]. also reported that the ΔADC% appears to be a reliable method to differentiate CR from non-CR patients after CRT in patients with LARC. In the current study, the ΔADC% in the pCR group was significantly greater than that in the non-pCR group (0.68 vs. 0.48, *p*<0.001), demonstrating the ΔADC% may be a predictor for pCR.

Besides the absolute pre-ADC value, the post-ADC value, and the ΔADC%, several studies had reported the cutoff value and predictive accuracy by the ROC curves. Kim et al.[20] had reported that the post-CRT ADC values in the pCR group were significantly higher than those in the non-pCR group, yielding a sensitivity of 100% and specificity of 84.6% for predicting pCR. While the pre-CRT ADC did not differ significantly between the pCR and the non-pCR patients. Lambrecht et al.[25] confirmed that the pre-CRT ADC value and the ΔADC% were significantly different in patients with a pCR compared to patients with a non-pCR. But the post-CRT ADC value could not reliably discriminate pCR from non-pCR patients. In a recent meta-analysis, Xie et al.[26] reported that the percentage increase of the ADC value had the highest diagnostic performance to predict a pCR compared with the pre-ADC and post-ADC.

In contrast to those previous studies, the current study has 50 pCR patients. We constructed the ROC curves for pCR prediction according to the pre-CRT ADC value and ΔADC%; the cut-off values were 0.866×10^-3^mm^2^/s and 58%, respectively. The curve showed that the AUC, sensitivity, specificity, and predictive accuracy of the pre-CRT ADC value were 0.670, 0.600, 0.640, and 60%, respectively. For the ΔADC%, they were 0.856, 0.800, 0.760, and 78%, respectively. It is analytically demonstrated that a pre-CRT ADC value that is below 0.866×10^-3^mm^2^/s and/or ΔADC% that is above 58% seems to be a significant indicator of the occurrence of pCR. We further compared the predicting efficacy and found that ΔADC% is a more reliable predictive indicator than the pre-CRT ADC value (*p*<0.001).

Magnetic resonance imaging (MRI), positron emission tomography-computed tomography (PET-CT), serum carcinoembryogenic antigen (CEA) levels, molecular biomarkers examined by immunohistochemistry, and gene expression profiling are the most useful and effective diagnostic methodologies for monitoring locally advanced rectal cancer. Diffusion-weighted MRI may be the best at detecting the dynamic changes of rectal cancer and for estimating the response at an early stage. Gene expression profiling and single nucleotide polymorphisms are considered to be promising to unveil the underlying complex genetics of the response to CRT. Due to the advantages and disadvantages of each, the multiple technologies are expected to be combined in the future to more accurately and more powerfully assess the response to NCRT [27,28].

The current study has several limitations. First, the number of patients in the study cohort is small and the design is a retrospective analysis. Second, this study matched control patients with similar conditions for analysis, which may reduce the experimental efficiency. Third, we observed and evaluated only two values before and after NCRT and we did not monitored all clinical components: Therefore, there is a certain one-sidedness to the study. In the future we will use a model with a random large sample and dynamic observations to make up for these deficiencies in the study.

## Conclusion

DWI-MRI technology can be efficacy to predict pCR for LARC after NCRT. Although the mean pre-CRT ADC value and the ΔADC% are moderate predictors for pCR, the latter one will be more accurate.

## Supporting Information

S1 Table Primary data of ADC for PlosOne. (XLS)
